# Diphenyl­methyl benzoate

**DOI:** 10.1107/S1600536812050064

**Published:** 2012-12-12

**Authors:** Manpreet Kaur, Jerry P. Jasinski, Amanda C. Keeley, H. S. Yathirajan, M. S. Siddegowda

**Affiliations:** a Department of Studies in Chemistry, University of Mysore, Manasagangotri, Mysore 570 006, India; b Department of Chemistry, Keene State College, 229 Main Street, Keene, NH 03435-2001, USA; c R. L. Fine Chem, Bengaluru, 560 064, India

## Abstract

In the title mol­ecule, C_20_H_16_O_2_, the dihedral angle between the phenyl rings of the diphenyl­methyl group is 68.3 (2)°. The benzoate group is essentially planar, with a maximum deviation of 0.017 (2) Å for the carbonyl O atom, and the two phenyl rings are twisted by 27.5 (4) and 85.6 (9)° from this plane. In the crystal, weak C—H⋯O hydrogen bonds link mol­ecules along [100].

## Related literature

For related structures, see: Baidya *et al.* (2009*a* ▶,*b* ▶); Gowda *et al.* (2007 ▶, 2009 ▶). For standard bond lengths, see: Allen *et al.* (1987 ▶).
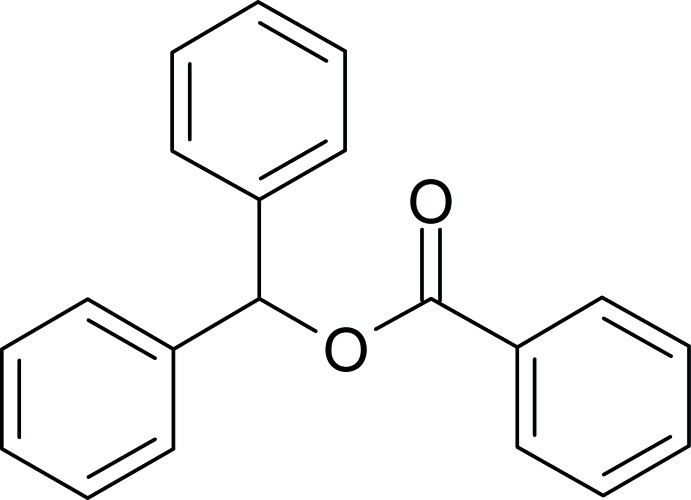


## Experimental

### 

#### Crystal data

C_20_H_16_O_2_
*M* *_r_* = 288.33
Monoclinic, 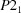

*a* = 5.75357 (19) Å
*b* = 16.0368 (5) Å
*c* = 8.3114 (3) Å
β = 95.340 (3)°
*V* = 763.55 (4) Å^3^
*Z* = 2
Cu *K*α radiation
μ = 0.63 mm^−1^
*T* = 173 K
0.38 × 0.26 × 0.24 mm

#### Data collection

Agilent Xcalibur (Eos, Gemini) diffractometer
Absorption correction: multi-scan (*CrysAlis PRO* and *CrysAlis RED*; Agilent, 2012 ▶) *T*_min_ = 0.912, *T*_max_ = 1.000
4414 measured reflections
2659 independent reflections
2528 reflections with *I* > 2σ(*I*)
*R*_int_ = 0.022

#### Refinement

*R*[*F* ^2^ > 2σ(*F* ^2^)] = 0.035
*wR*(*F* ^2^) = 0.094
*S* = 1.06
2659 reflections
200 parameters
1 restraint
H-atom parameters constrained
Δρ_max_ = 0.21 e Å^−3^
Δρ_min_ = −0.15 e Å^−3^
Absolute structure: Flack (1983 ▶) 1120 Friedel pairs
Flack parameter: 0.0 (2)

### 

Data collection: *CrysAlis PRO* (Agilent, 2012 ▶); cell refinement: *CrysAlis PRO*; data reduction: *CrysAlis RED* (Agilent, 2012 ▶); program(s) used to solve structure: *SHELXS97* (Sheldrick, 2008 ▶); program(s) used to refine structure: *SHELXL97* (Sheldrick, 2008 ▶); molecular graphics: *SHELXTL* (Sheldrick, 2008 ▶); software used to prepare material for publication: *SHELXTL*.

## Supplementary Material

Crystal structure: contains datablock(s) global, I. DOI: 10.1107/S1600536812050064/lh5566sup1.cif

Structure factors: contains datablock(s) I. DOI: 10.1107/S1600536812050064/lh5566Isup2.hkl

Supplementary material file. DOI: 10.1107/S1600536812050064/lh5566Isup3.cml

Additional supplementary materials:  crystallographic information; 3D view; checkCIF report

## Figures and Tables

**Table 1:** Hydrogen-bond geometry (Å, °)

*D*—H⋯*A*	*D*—H	H⋯*A*	*D*⋯*A*	*D*—H⋯*A*
C16—H16⋯O2^i^	0.93	2.44	3.334 (2)	160

Symmetry code: (i) 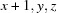.

**Fig. 1.**
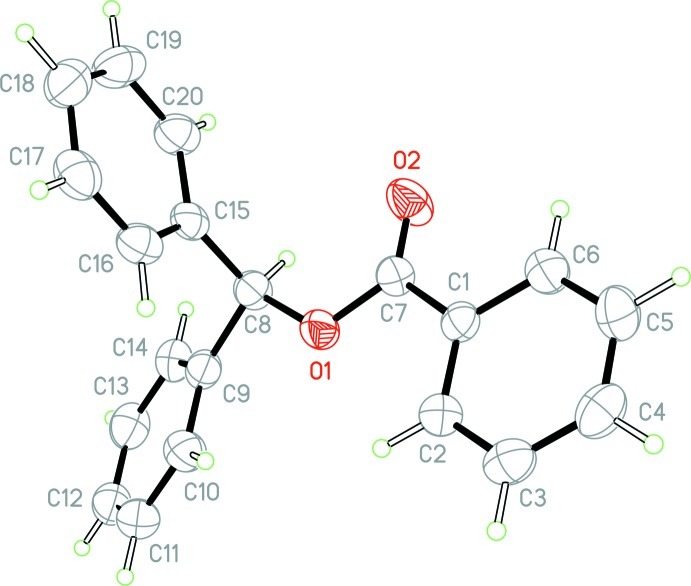
Molecular structure of the title compound showing 50% probability displacement ellipsoids.

**Fig. 2.**
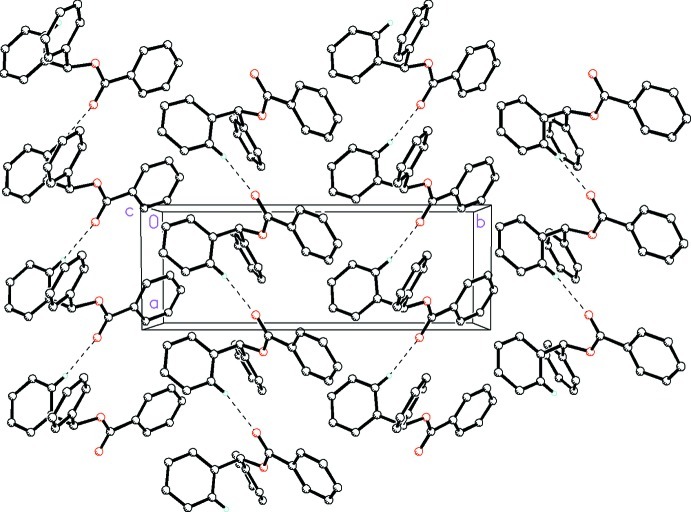
Packing diagram of the title compound viewed along the c axis showing weak C—H···O intermolecular interactions (dashed lines) linking the molecules into columns along [100]

## supplementary crystallographic information

### Comment

Benzyl Benzoate is widely used in the perfume and pharmaceutical industries. The crystal structures of some related compounds, viz., 4,4'-bis(dimethylamino)benzhydryl phenyl sulfone (Baidya *et al.*, 2009*a*), benzhydryl phenyl sulfone (Baidya *et al.*, 2009*b*), 4-methylphenyl benzoate (Gowda *et al.*, 2007), 2,4-dimethylphenyl 4-methylbenzoate (Gowda *et al.*, 2009) have been reported. In view of the importance of benzoates, the paper reports the crystal structure of the title compound, (I).

The molecular structure of the title compound is shown in Fig. 1. The dihedral angle between the two phenyl rings (C9-C14 and C15-C20) is 68.3 (2)°. The mean plane of the benzoate group (C1–C7/O1/O2, with a maximum deviation of 0.017 (2)Å for O2) is twisted by 27.5 (4)° (C9–C14) and 85.6 (9)° (C15–C20), respectively, from that of the phenyl rings. In the crystal, weak C—H···O hydrogen bonds (Table 1) link molecules along [100] (Fig. 2).

### Experimental

The title compound was obtained as a gift sample from R. L. Fine Chem, Bengaluru, India. X-ray quality crystals were obtained by slow evaporation of acetone and acetone solution (m.p.: 353–355 K).

### Refinement

All of the H atoms were placed in their calculated positions and then refined using the riding model with Atom—H lengths of 0.93Å (CH). Isotropic displacement parameters for these atoms were set to 1.19-1.20 (CH) times *U*_eq_ of the parent atom.

### Crystal data

**Table:**

C_20_H_16_O_2_	*F*(000) = 304
*M**_r_* = 288.33	*D*_x_ = 1.254 Mg m^−^^3^
Monoclinic, *P*2_1_	Cu *K*α radiation, λ = 1.54184 Å
Hall symbol: P 2yb	Cell parameters from 2446 reflections
*a* = 5.75357 (19) Å	θ = 5.3–72.4°
*b* = 16.0368 (5) Å	µ = 0.63 mm^−^^1^
*c* = 8.3114 (3) Å	*T* = 173 K
β = 95.340 (3)°	Block, colorless
*V* = 763.55 (4) Å^3^	0.38 × 0.26 × 0.24 mm
*Z* = 2	

### Data collection

**Table:**

Agilent Xcalibur (Eos, Gemini) diffractometer	2659 independent reflections
Radiation source: Enhance (Cu) X-ray Source	2528 reflections with *I* > 2σ(*I*)
Graphite monochromator	*R*_int_ = 0.022
Detector resolution: 16.0416 pixels mm^-1^	θ_max_ = 72.5°, θ_min_ = 5.4°
ω scans	*h* = −7→5
Absorption correction: multi-scan (*CrysAlis PRO* and *CrysAlis RED*; Agilent, 2012)	*k* = −17→19
*T*_min_ = 0.912, *T*_max_ = 1.000	*l* = −6→10
4414 measured reflections	

### Refinement

**Table:**

Refinement on *F*^2^	Hydrogen site location: inferred from neighbouring sites
Least-squares matrix: full	H-atom parameters constrained
*R*[*F*^2^ > 2σ(*F*^2^)] = 0.035	*w* = 1/[σ^2^(*F*_o_^2^) + (0.0518*P*)^2^ + 0.052*P*] where *P* = (*F*_o_^2^ + 2*F*_c_^2^)/3
*wR*(*F*^2^) = 0.094	(Δ/σ)_max_ < 0.001
*S* = 1.06	Δρ_max_ = 0.21 e Å^−^^3^
2659 reflections	Δρ_min_ = −0.15 e Å^−^^3^
200 parameters	Extinction correction: *SHELXL97* (Sheldrick, 2008), Fc^*^=kFc[1+0.001xFc^2^λ^3^/sin(2θ)]^-1/4^
1 restraint	Extinction coefficient: 0.0104 (11)
Primary atom site location: structure-invariant direct methods	Absolute structure: Flack (1983) 1120 Friedel pairs
Secondary atom site location: difference Fourier map	Flack parameter: 0.0 (2)

### Special details

**Table:**

Geometry. All esds (except the esd in the dihedral angle between two l.s. planes) are estimated using the full covariance matrix. The cell esds are taken into account individually in the estimation of esds in distances, angles and torsion angles; correlations between esds in cell parameters are only used when they are defined by crystal symmetry. An approximate (isotropic) treatment of cell esds is used for estimating esds involving l.s. planes.
Refinement. Refinement of *F*^2^ against ALL reflections. The weighted *R*-factor *wR* and goodness of fit *S* are based on *F*^2^, conventional *R*-factors *R* are based on *F*, with *F* set to zero for negative *F*^2^. The threshold expression of *F*^2^ > σ(*F*^2^) is used only for calculating *R*-factors(gt) *etc*. and is not relevant to the choice of reflections for refinement. *R*-factors based on *F*^2^ are statistically about twice as large as those based on *F*, and *R*- factors based on ALL data will be even larger.

### Fractional atomic coordinates and isotropic or equivalent isotropic displacement parameters (Å^2^)

**Table:**

	*x*	*y*	*z*	*U*_iso_*/*U*_eq_	
O1	0.2173 (2)	0.33454 (8)	0.50213 (15)	0.0376 (3)	
O2	−0.1042 (2)	0.31628 (10)	0.63104 (18)	0.0495 (4)	
C1	0.1598 (3)	0.42599 (10)	0.7144 (2)	0.0312 (4)	
C2	0.3646 (3)	0.46769 (11)	0.6891 (2)	0.0364 (4)	
H2	0.4547	0.4498	0.6085	0.044*	
C3	0.4349 (4)	0.53567 (12)	0.7833 (2)	0.0434 (4)	
H3	0.5709	0.5640	0.7648	0.052*	
C4	0.3036 (4)	0.56172 (13)	0.9049 (2)	0.0465 (5)	
H4	0.3514	0.6074	0.9685	0.056*	
C5	0.1008 (4)	0.51969 (13)	0.9320 (2)	0.0491 (5)	
H5	0.0133	0.5370	1.0145	0.059*	
C6	0.0279 (3)	0.45254 (12)	0.8375 (2)	0.0407 (4)	
H6	−0.1092	0.4248	0.8556	0.049*	
C7	0.0735 (3)	0.35370 (11)	0.6150 (2)	0.0329 (4)	
C8	0.1615 (3)	0.26034 (11)	0.4044 (2)	0.0339 (4)	
H8	−0.0074	0.2579	0.3758	0.041*	
C9	0.2833 (3)	0.27104 (10)	0.2523 (2)	0.0350 (4)	
C10	0.4936 (3)	0.31388 (13)	0.2513 (2)	0.0408 (4)	
H10	0.5615	0.3381	0.3459	0.049*	
C11	0.6021 (4)	0.32051 (14)	0.1099 (3)	0.0459 (5)	
H11	0.7420	0.3495	0.1101	0.055*	
C12	0.5040 (4)	0.28435 (12)	−0.0315 (2)	0.0452 (5)	
H12	0.5776	0.2890	−0.1261	0.054*	
C13	0.2964 (4)	0.24132 (14)	−0.0317 (2)	0.0459 (5)	
H13	0.2304	0.2166	−0.1264	0.055*	
C14	0.1859 (3)	0.23482 (12)	0.1092 (2)	0.0400 (4)	
H14	0.0455	0.2060	0.1081	0.048*	
C15	0.2380 (3)	0.18357 (11)	0.50053 (19)	0.0327 (4)	
C16	0.4629 (3)	0.17795 (12)	0.5773 (2)	0.0381 (4)	
H16	0.5683	0.2212	0.5672	0.046*	
C17	0.5304 (4)	0.10861 (14)	0.6684 (2)	0.0456 (5)	
H17	0.6804	0.1056	0.7204	0.055*	
C18	0.3756 (4)	0.04356 (13)	0.6827 (2)	0.0494 (5)	
H18	0.4206	−0.0028	0.7452	0.059*	
C19	0.1538 (4)	0.04782 (13)	0.6037 (3)	0.0514 (5)	
H19	0.0504	0.0038	0.6115	0.062*	
C20	0.0851 (4)	0.11732 (13)	0.5130 (2)	0.0421 (4)	
H20	−0.0644	0.1197	0.4601	0.051*	

### Atomic displacement parameters (Å^2^)

**Table:**

	*U*^11^	*U*^22^	*U*^33^	*U*^12^	*U*^13^	*U*^23^
O1	0.0385 (7)	0.0341 (7)	0.0422 (7)	−0.0057 (5)	0.0136 (5)	−0.0087 (5)
O2	0.0401 (7)	0.0565 (9)	0.0543 (8)	−0.0152 (6)	0.0172 (6)	−0.0205 (7)
C1	0.0333 (8)	0.0287 (8)	0.0316 (8)	0.0040 (6)	0.0032 (6)	0.0031 (6)
C2	0.0354 (9)	0.0348 (9)	0.0396 (9)	−0.0008 (7)	0.0058 (7)	0.0002 (7)
C3	0.0415 (10)	0.0386 (10)	0.0494 (11)	−0.0062 (8)	0.0002 (8)	0.0021 (9)
C4	0.0569 (12)	0.0358 (10)	0.0451 (10)	−0.0034 (9)	−0.0047 (9)	−0.0076 (8)
C5	0.0602 (13)	0.0475 (12)	0.0410 (11)	0.0031 (10)	0.0129 (9)	−0.0103 (9)
C6	0.0410 (10)	0.0402 (10)	0.0419 (10)	−0.0016 (8)	0.0082 (8)	−0.0030 (8)
C7	0.0318 (8)	0.0333 (9)	0.0340 (8)	0.0016 (7)	0.0047 (7)	−0.0001 (7)
C8	0.0327 (8)	0.0345 (9)	0.0349 (8)	−0.0039 (7)	0.0050 (7)	−0.0062 (7)
C9	0.0395 (9)	0.0303 (9)	0.0354 (8)	0.0045 (7)	0.0056 (7)	0.0010 (7)
C10	0.0428 (10)	0.0413 (10)	0.0393 (9)	−0.0018 (8)	0.0088 (8)	−0.0016 (8)
C11	0.0495 (11)	0.0415 (11)	0.0487 (10)	0.0004 (9)	0.0152 (8)	0.0058 (9)
C12	0.0589 (12)	0.0427 (11)	0.0363 (9)	0.0099 (9)	0.0172 (9)	0.0070 (8)
C13	0.0609 (12)	0.0443 (11)	0.0324 (9)	0.0086 (9)	0.0032 (8)	−0.0021 (8)
C14	0.0420 (9)	0.0379 (10)	0.0401 (9)	0.0039 (8)	0.0035 (8)	−0.0035 (8)
C15	0.0374 (9)	0.0340 (9)	0.0282 (8)	−0.0040 (7)	0.0108 (7)	−0.0075 (6)
C16	0.0391 (10)	0.0423 (10)	0.0338 (9)	−0.0060 (8)	0.0076 (7)	−0.0023 (7)
C17	0.0448 (11)	0.0580 (13)	0.0349 (9)	0.0062 (9)	0.0083 (8)	0.0008 (9)
C18	0.0722 (15)	0.0392 (11)	0.0382 (10)	0.0059 (10)	0.0123 (9)	0.0006 (8)
C19	0.0683 (14)	0.0364 (11)	0.0504 (11)	−0.0152 (10)	0.0108 (10)	−0.0032 (9)
C20	0.0421 (10)	0.0423 (10)	0.0427 (10)	−0.0100 (8)	0.0073 (8)	−0.0065 (8)

### Geometric parameters (Å, º)

**Table:**

O1—C7	1.343 (2)	C10—C11	1.385 (3)
O1—C8	1.460 (2)	C10—H10	0.9300
O2—C7	1.203 (2)	C11—C12	1.383 (3)
C1—C2	1.388 (2)	C11—H11	0.9300
C1—C6	1.396 (2)	C12—C13	1.379 (3)
C1—C7	1.482 (2)	C12—H12	0.9300
C2—C3	1.381 (3)	C13—C14	1.387 (3)
C2—H2	0.9300	C13—H13	0.9300
C3—C4	1.382 (3)	C14—H14	0.9300
C3—H3	0.9300	C15—C20	1.390 (2)
C4—C5	1.384 (3)	C15—C16	1.392 (2)
C4—H4	0.9300	C16—C17	1.380 (3)
C5—C6	1.375 (3)	C16—H16	0.9300
C5—H5	0.9300	C17—C18	1.384 (3)
C6—H6	0.9300	C17—H17	0.9300
C8—C15	1.511 (3)	C18—C19	1.381 (3)
C8—C9	1.511 (2)	C18—H18	0.9300
C8—H8	0.9800	C19—C20	1.382 (3)
C9—C10	1.392 (3)	C19—H19	0.9300
C9—C14	1.394 (3)	C20—H20	0.9300
			
C7—O1—C8	117.21 (13)	C11—C10—H10	119.9
C2—C1—C6	119.42 (17)	C9—C10—H10	119.9
C2—C1—C7	122.53 (15)	C12—C11—C10	120.54 (19)
C6—C1—C7	118.05 (16)	C12—C11—H11	119.7
C3—C2—C1	120.18 (18)	C10—C11—H11	119.7
C3—C2—H2	119.9	C13—C12—C11	119.72 (17)
C1—C2—H2	119.9	C13—C12—H12	120.1
C2—C3—C4	120.15 (18)	C11—C12—H12	120.1
C2—C3—H3	119.9	C12—C13—C14	120.08 (18)
C4—C3—H3	119.9	C12—C13—H13	120.0
C3—C4—C5	119.89 (18)	C14—C13—H13	120.0
C3—C4—H4	120.1	C13—C14—C9	120.65 (18)
C5—C4—H4	120.1	C13—C14—H14	119.7
C6—C5—C4	120.37 (19)	C9—C14—H14	119.7
C6—C5—H5	119.8	C20—C15—C16	118.97 (17)
C4—C5—H5	119.8	C20—C15—C8	120.50 (16)
C5—C6—C1	119.98 (18)	C16—C15—C8	120.53 (16)
C5—C6—H6	120.0	C17—C16—C15	120.40 (18)
C1—C6—H6	120.0	C17—C16—H16	119.8
O2—C7—O1	123.26 (16)	C15—C16—H16	119.8
O2—C7—C1	124.91 (16)	C16—C17—C18	120.2 (2)
O1—C7—C1	111.83 (14)	C16—C17—H17	119.9
O1—C8—C15	109.39 (13)	C18—C17—H17	119.9
O1—C8—C9	106.13 (14)	C19—C18—C17	119.7 (2)
C15—C8—C9	113.59 (14)	C19—C18—H18	120.1
O1—C8—H8	109.2	C17—C18—H18	120.1
C15—C8—H8	109.2	C18—C19—C20	120.2 (2)
C9—C8—H8	109.2	C18—C19—H19	119.9
C10—C9—C14	118.75 (16)	C20—C19—H19	119.9
C10—C9—C8	122.17 (15)	C19—C20—C15	120.42 (19)
C14—C9—C8	119.06 (16)	C19—C20—H20	119.8
C11—C10—C9	120.26 (18)	C15—C20—H20	119.8
			
C6—C1—C2—C3	−1.1 (3)	C14—C9—C10—C11	0.4 (3)
C7—C1—C2—C3	178.80 (17)	C8—C9—C10—C11	178.47 (18)
C1—C2—C3—C4	1.0 (3)	C9—C10—C11—C12	−0.4 (3)
C2—C3—C4—C5	−0.2 (3)	C10—C11—C12—C13	0.0 (3)
C3—C4—C5—C6	−0.6 (3)	C11—C12—C13—C14	0.4 (3)
C4—C5—C6—C1	0.5 (3)	C12—C13—C14—C9	−0.4 (3)
C2—C1—C6—C5	0.3 (3)	C10—C9—C14—C13	0.0 (3)
C7—C1—C6—C5	−179.60 (17)	C8—C9—C14—C13	−178.14 (17)
C8—O1—C7—O2	−5.1 (3)	O1—C8—C15—C20	129.89 (16)
C8—O1—C7—C1	175.31 (13)	C9—C8—C15—C20	−111.75 (18)
C2—C1—C7—O2	−179.04 (19)	O1—C8—C15—C16	−50.40 (19)
C6—C1—C7—O2	0.8 (3)	C9—C8—C15—C16	68.0 (2)
C2—C1—C7—O1	0.5 (2)	C20—C15—C16—C17	−1.9 (3)
C6—C1—C7—O1	−179.61 (16)	C8—C15—C16—C17	178.38 (15)
C7—O1—C8—C15	−78.53 (17)	C15—C16—C17—C18	0.7 (3)
C7—O1—C8—C9	158.56 (14)	C16—C17—C18—C19	0.8 (3)
O1—C8—C9—C10	31.5 (2)	C17—C18—C19—C20	−1.1 (3)
C15—C8—C9—C10	−88.7 (2)	C18—C19—C20—C15	−0.1 (3)
O1—C8—C9—C14	−150.37 (15)	C16—C15—C20—C19	1.6 (3)
C15—C8—C9—C14	89.40 (19)	C8—C15—C20—C19	−178.71 (17)

### Hydrogen-bond geometry (Å, º)

**Table:**

*D*—H···*A*	*D*—H	H···*A*	*D*···*A*	*D*—H···*A*
C16—H16···O2^i^	0.93	2.44	3.334 (2)	160

Symmetry code: (i) *x*+1, *y*, *z*.
